# The Fabrication of Porous Si with Interconnected Micro-Sized Dendrites and Tunable Morphology through the Dealloying of a Laser Remelted Al–Si Alloy

**DOI:** 10.3390/ma10040357

**Published:** 2017-03-28

**Authors:** Ting Huang, Dingyue Sun, Wuxiong Yang, Qiang Wu, Rongshi Xiao

**Affiliations:** High-Power and Ultrafast Laser Manufacturing Laboratory, Institute of Laser Engineering, Beijing University of Technology, Beijing 100124, China; sundingyue@emails.bjut.edu.cn (D.S.); bbgei@bjut.edu.cn (W.Y.); jlwuqiang@bjut.edu.cn (Q.W.); rsxiao@bjut.edu.cn (R.X.)

**Keywords:** porous materials, laser processing, dealloying, silicon

## Abstract

Coral-like porous Si was fabricated through the dealloying of a laser remelted as-cast AlSi12 alloy(Al-12 wt % Si). The porous Si was composed of interconnected micro-sized Si dendrites and micro/nanopores, and compared to flaky Si, which is fabricated by direct dealloying of the as-cast AlSi12 alloy. The structure of the porous Si was attributed to the dendritic solidification microstructure formed during the laser remelting process. The micropore size of the porous Si decreased from 4.2 μm to 1.6 µm with the increase in laser scanning velocity, indicating that the morphology of porous Si could be easily altered by simply controlling the laser remelting parameters. The coral-like porous Si provided enough space, making it promising for high-performance Si-based composite anode materials in lithium-ion batteries. The proposed hybrid method provides a straightforward way of tuning the porous structure in the dealloyed material.

## 1. Introduction

Silicon (Si) is regarded as the most promising anode material for high-performance lithium-ion batteries (LIBs) owing to its outstanding volumetric/gravimetric capacity and abundance [1,2,3]. However, the use of bulk Si is impeded by the well-known pulverization of Si due to inhomogeneous volume expansion (~300%) during lithiation and delithiation cycles [4,5,6].

To address this problem, one successful method is to fabricate micro-sized porous Si–C composites by coating carbon onto micro-sized porous Si [7]. Tian et al. [8] and Yi et al. [9] showed that the micro-sized porous Si–C composites could greatly improve electrochemical performance with high capacity and good cycling stability. The enhanced electrochemical performance is attributed to the following factors: (a) the micro-sized porous Si takes advantages of both high volumetric/gravimetric capacity of micro-Si and adequate space provided by the inside pore for Si volume expansion; and (b) the electrical contact and stability is improved by the carbon coating. Therefore, fabricating the micro-sized porous Si is the premise to obtain the micro-sized porous Si–C composites.

Compared to chemical vapor deposition [10,11], magnesium reduction [12,13] and vaporization [14], etching is a simple and efficient method of fabricating porous Si [15,16,17,18]. As carbon-coated porous Si retains structural characteristics of as-fabricated porous Si, properties of Si–C composites can be improved by turning the structure of porous Si. The structure of porous Si fabricated by etching is largely determined by two factors, i.e., the etching conditions, and the microstructure of pristine material prior to etching. Bang et al. [19] showed that morphologies of the as-synthesized porous Si could be tuned by controlling the etching conditions. In comparison, there is a lack of research on the development of methods for the microstructural modification of pristine material. Here, a laser remelting-selective etching (also known as dealloying) hybrid method for the fabrication of porous Si with tunable morphology is presented. The fabrication method is introduced, and the effect of laser remelting on the formation of the porous Si is explained.

## 2. Materials and Methods

**Figure 1:**
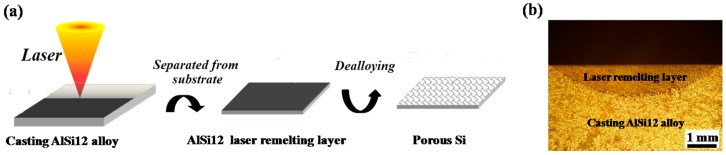
(**a**) Schematic illustration showing the laser remelting-dealloying fabrication process and (**b**) cross section of laser remelting layer (5.5 kW, 5 mm/s).

Figure 1a shows the schematic illustration of the laser remelting-dealloying fabrication process. A continuous-wave fiber laser (wavelength 1070 nm, IPG YLS-6000, IPG Photonics Corporation, Oxford, UK) with a beam diameter of 6 mm was used to scan over the commercial casting AlSi12 alloy (Al-12 wt % Si) plate to form a laser remelting layer, which was then cut from the AlSi12 substrate and immersed into 3 M HCl solution for 4 h to selectively remove Al as part of the dealloying process. During laser remelting process, single-track laser remelting was performed with the laser power of 5.5 kW, while argon was blown to the melted surface to prevent oxidation at a flowing rate of 18 L/min. In order to investigate the effects of laser remelting on subsequent dealloying, individual laser remelting layers were fabricated at laser beam scanning velocity of 5 mm/s, 7 mm/s, 9 mm/s and 11 mm/s. Figure 1b shows the typical cross section of the laser remelting layer. The width and thickness of the laser remelting layer was determined by the laser scanning velocity. The dimension of each specimen for subsequent dealloying was 2 mm long, 2 mm wide and 150 μm thick. Microstructures of initial as-cast AlSi12 alloy, laser remelting layer, and final porous Si were examined using a scanning electron microscope (SEM, HITACHI S-3400, Hitachi, Ltd., Tokyo, Japan) equipped with energy-dispersive X-ray spectroscopy (EDS). Cross section of laser remelting layer was examined by optical microscopy (OLYMPUS, GX51, Olympus Corporation, Tokyo, Japan). X-ray diffraction (XRD) patterns were recorded by an X-ray diffractometer (BRUKER D8 Advance, Cu target, Bruker Corporation, Karlsruhe, Germany) for phase analysis.

## 3. Results and Discussion

**Figure 2:**
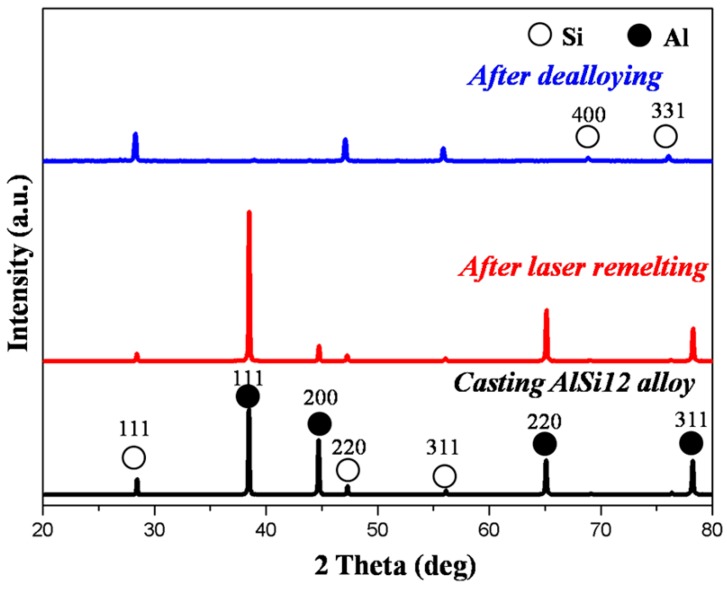
XRD patterns of the initial casting AlSi12 alloy and the laser remelting layer showing Si and Al. XRD patterns after dealloying showing Si.

Figure 2 shows the XRD patterns of the initial casting AlSi12 alloy, the laser remelting layer fabricated at laser beam scanning velocity of 7 mm/s, and the final structure after dealloying. In the casting AlSi12 alloy and the laser remelting layer, Al and Si phases were indexed (JCPDS No. 27-1402). No clear phase transformation happened during the laser remelting process. After dealloying process, only Si was indentified, demonstrating the selective etching of Al from AlSi12 alloy during dealloying process.

**Figure 3:**
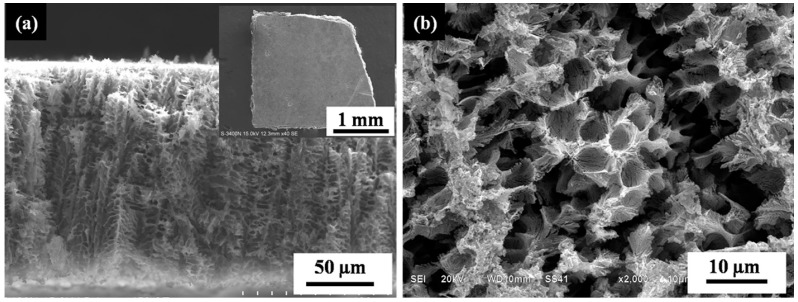

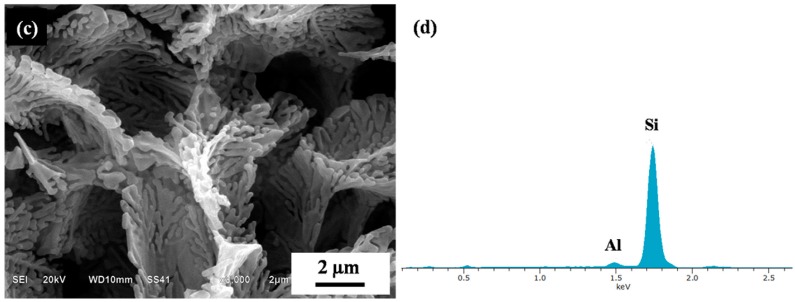
Microstructure characterization of the porous Si: (**a**) SEM image of the crosssection of the porous Si (inset is top-view macrophotograph); (**b**,**c**) top-view SEM images of the porous Si and (**d**) EDS result.

A typical resulting structure after dealloying is shown in Figure 3. Figure 3a is a cross-sectional image of porous Si. The thickness of the porous Si was 150 μm, which was in accordance with the thickness of the laser remelting layer, indicating that the laser remelting layer was completely etched during dealloying. The top-view SEM image shows that the porous Si exhibited a coral-like structure, which was composed of Si walls and external micro-sized pores surrounded by Si walls (Figure 3b). At a higher magnification (Figure 3c), finer substructures could be clearly observed on the surface of the Si walls, which were identified as continues networks consisting of interconnected micro-sized Si dendrites and internal nano-sized pores. It is noted that Al-94.3 wt % Si was identified by EDS in the porous Si (Figure 3d). The absence of Al in XRD result may due to its low proportion. The as-fabricated coral-like structure with continues networks conceivably presents great benefits for high-performance Si-based composite anode materials in LIBs. Kim et al. [20] showed that the interconnected structure improved the cycling stability, and the external and internal pores provided enough space for volume change alleviation and fast Li diffusion. Conventionally, the porous Si is fabricated in the form of micro-sized particles. The porous Si-based anodes of LIBs are prepared by casting mixed slurry of micro-sized porous Si particles, conductive additive and binder onto copper foil [8,9,15]. However, the mixed slurry lacks a well-interconnected pore, which undesirably reduces the space for volume change of Si and the contact between electrolyte and Si [19]. Moreover, the addition of conductive additive and binder dilutes the loading mass of Si, which impeded its industrial applications [21]. In the present study, the large-scale interconnected porous Si was achieved (Figure 3a inset), potentiating its practical applications due to the absence of binder and the increased loading mass of Si.

**Figure 4:**
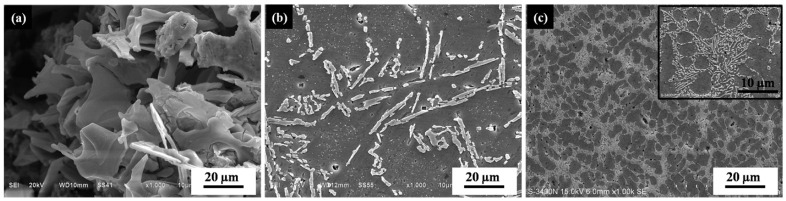
Top-view SEM images showing (**a**) flaky Si fabricated by dealloying of the as-cast AlSi12 alloy (without laser remelting); (**b**) microstructure of the as-cast AlSi12 alloy and (**c**) microstructure of the laser remelting layer.

In contrast, flaky Si (Figure 4a) was obtained through direct dealloying of as-cast AlSi12 alloy (without laser remelting process). The results demonstrated that the laser remelting process enabled the formation of coral-like porous Si. Comparisons between casting and laser remelted microstructure permit to understand the laser effects. The Al–Si alloy is known as a eutectic system in which a eutectic reaction occurs when Si content is in a range of 11–13% by weight. For AlSi12 casting, the eutectic microstructure consisted of Al matrix and the separated distribution of needle-shape Si (Figure 4b). In comparison, the dendritic microstructure was observed in the laser remelting layer (Figure 4c). The dark and white regions are primary α-Al dendritic matrix and interdendritic (α-Al+Si) eutectic, respectively (Figure 4c inset). In general, laser remelting significantly influences the solidification microstructure due to a very high cooling rate (10^5^ to 10^8^ K/s) with an ultra-high temperature gradient in the remelted surface [22,23,24]. The α-Al dendritic with interdendritic (α-Al+Si) eutectic obtained by laser remelting indicated that the solidification occurred outside the coupled zone in the Al–Si system, even for AlSi12 with eutectic composition, resulted from the increased undercooling. Further, cooling of the laser remelted surface mostly occurred via the substrate, leading to the directional heat flow toward the substrate. Thus, the α-Al columnar dendritic was perpendicular to the solid/liquid interface, which was oriented opposite to the heat-flow direction. Based on the microstructure observation, we suggest that the external pores perpendicular to the substrate and the Si walls with internal pores in the coral-like porous Si are derived from the primary α-Al matrix and interdendritic (α-Al+Si) eutectic after the removing of Al, respectively. Generally speaking, the homogeneous composition of precursor alloy prior to dealloying is a prerequisite for the formation of porous structures [25]. However, in the laser remelting layer, the dendritic core and the interdendritic region had different compositions. The results show that an inhomogeneous alloy with phase separation can be exploited to fabricate alternative porous structures. Therefore, our proposed hybrid method can be readily extended to fabricate of a wide range of porous structures. For example, nanoporous Mn was obtained through the dealloying of laser processed Mn-Cu alloy with dendritic microstructure in our previous studies [26,27].

**Figure 5:**
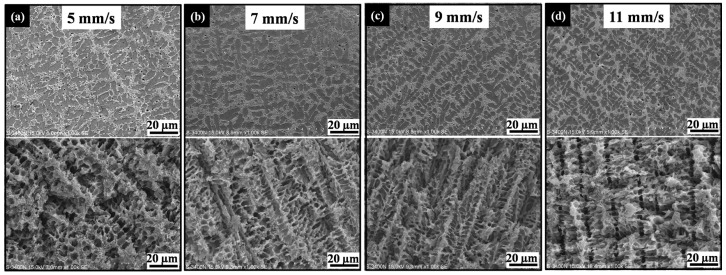
SEM images showing the effects of laser scanning velocity on microstructure of laser remelting layer and morphology of porous Si: (**a**) 5 mm/s; (**b**) 7 mm/s; (**c**) 9 mm/s; (**d**) 11 mm/s.

By observing the laser remelted samples at a different laser scanning velocity before and after dealloying, a series of microstructures of the laser remelting layer and morphology of porous Si was obtained. Figure 4 shows that the dendritic microstructure was achieved in all samples undergoing a laser scanning velocity of 5 mm/s (Figure 5a), 7 mm/s (Figure 5b), 9 mm/s (Figure 5c), and 11 mm/s (Figure 5d). In contrast, increasing the laser scanning velocity decreased the dendrite arm spacing and eutectic spacing, which strongly depend on the cooling rate of the solidification process. As a result, the external pore size of the porous Si decreased from 4.2 µm (5 mm/s) to 1.6 µm (11 mm/s) with the increase in laser scanning velocity. The results indicate that the pore size can be easily altered by simply controlling the laser remelting parameters.

## 4. Conclusions

In conclusion, coral-like porous Si with a tunable morphology was achieved by a hybrid method involving laser remelting followed by dealloying. The porous Si was composed of interconnected micro-sized Si dendrites and pores surrounded by Si dendrites, making it promising for high-performance Si-based composite anode materials in LIBs. The dendritic microstructure by laser remelting enabled the formation of porous Si. The proposed hybrid method provides a straightforward way of tuning the porous structure in the dealloyed material.
